# Measuring carbonic anhydrase IX as a hypoxia biomarker: differences in concentrations in serum and plasma using a commercial enzyme-linked immunosorbent assay due to influences of metal ions

**DOI:** 10.1258/acb.2010.010240

**Published:** 2011-03

**Authors:** Tobias C Wind, Michael P Messenger, Douglas Thompson, Peter J Selby, Rosamonde E Banks

**Affiliations:** 1 Clinical and Biomedical Proteomics Group, Cancer Research UK Centre, Leeds Institute of Molecular Medicine, St James's University Hospital; 2 Leeds Teaching Hospitals NHS Trust, St James's University Hospital, Beckett Street, Leeds, UK

## Abstract

**Background:**

There is increasing interest in measuring the soluble forms of carbonic anhydrase IX (CA IX) in blood as a marker of hypoxia for prognostic purposes or for predictive use in therapeutic trials in various cancers. Following our initial observations of marked differences in the measured concentrations of CA IX in EDTA plasma versus serum, we sought to investigate these further in order to determine their effects on results in published studies and to ensure accurate measurement in future studies.

**Methods:**

Serum and EDTA plasma samples from healthy controls and patients with renal cancer were used in the validation of two commercially available enzyme-linked immunosorbent assays (ELISAs) for CA IX with examination of recovery, parallelism and specificity and comparison of paired plasma and serum.

**Results:**

Successful validation of one of the ELISAs was not achieved with particular problems with parallelism and marked differences in measured CA IX concentrations between EDTA plasma and serum. This appeared to be due to a metal ion-dependent epitope on CA IX recognized by the detection antibody in this assay. The other commercially available ELISA examined was successfully validated and showed no difference in CA IX between EDTA plasma and serum.

**Conclusions:**

These results have important consequences for published studies using this assay where the conclusions drawn from the measurements made may be invalid. This study highlights the need for stringent validation of commercially available assays, including examination of various sample types, before use in research studies.

## Introduction

The membrane protein carbonic anhydrase IX (CA IX) plays a key role in maintaining cellular pH through catalysing the reversible hydration of carbon dioxide to H^+^ and HCO_3_-, allowing cell survival in hypoxic anaerobic conditions for example.^1^ Aberrant expression in cancer was initially reported in 1986 with the reactivity of an antibody clone (G250) with the cell membrane of renal cell carcinoma cells (RCCs) but not normal renal tissue^2^ and subsequent identification of the relevant antigen as CA IX/MN protein.^3,4^ With a predicted molecular weight of 46 kDa, several forms exist through alternative splicing, proteolytic cleavage, glycosylation and phosphorylation.^5–8^

CA IX is up-regulated in several cancers^9^ due to its regulation by hypoxia-inducible factor-1*α* (HIF-1*α*), a key transcriptional regulator of genes involved in the response to hypoxia.^10^ In sporadic conventional (clear cell) RCCs, the involvement of the Von Hippel Lindau tumour suppressor gene in most cases^11^ also leads to increased expression of HIF-1*α* and consequently proteins such as CA IX.^12,13^ With its limited expression in normal tissues,^2,7,10^ CA IX is being exploited as a potential therapeutic target using several strategies including antibody-targeting, vaccine-based or small molecule inhibitors.^14–17^

The potential role of CA IX as a biomarker in RCCs has been examined in diagnostic imaging using G250.^18^ Several immunohistochemical studies have shown the prognostic significance of CA IX tissue expression, particularly in patients with metastatic disease with low expression being associated with poorer outcome, although there is disagreement between studies.^9,16^ Several initial studies have also now begun to examine potential clinical utility of soluble forms of CA IX in the serum or plasma from patients with RCCs and also other cancers^19–27^ both for prognostic and predictive applications with some interesting but conflicting findings. While validating a commercial enzyme-linked immunosorbent assay (ELISA) for use in a prognostic study in RCCs, we found marked differences between the detectable concentrations of CA IX depending on whether serum or EDTA plasma was used and report here the subsequent investigations which highlight some important considerations when measuring soluble CA IX.

## Materials and methods

### Samples

Matched serum and EDTA plasma samples from 17 patients with RCCs of varying stage and grade (10 men, 7 women; age range, 42–75 y) and 10 healthy controls (4 men, 6 women; age range 21–66 y) were selected for this study. Different but overlapping subsets of these samples were used for the various parts of the assay validation as indicated in each area. All samples had been obtained in Leeds during 2006–2010 with informed consent and following approval by a local research ethics committee. Venepuncture was carried out using the Vacuette^®^ system (Greiner Bio-One, Frickenhausen, Germany), using Z/serum clot-activator tubes (coated with micronized silica particles) and EDTA plasma tubes (coated with 1.8 mg/mL K_2_EDTA). Samples were maintained at room temperature (20°C) postvenepuncture for 45 min before centrifugation at 2000***g*** at 20°C for 10 min. Serum and plasma were aspirated, aliquoted and stored at −80°C until used.

### CA IX ELISAs

Initially the commercially available human MN/CA IX ELISA kit from Oncogene Science/Siemens Healthcare Diagnostics Inc. (Cambridge, MA, USA) with a stated use of either serum or plasma (citrate, heparin or EDTA) was the main focus of the validation, with our intended use being with EDTA plasma samples. However, following some of the initial findings, validation and sample analysis was also undertaken using the Quantikine human CA IX/CA9 ELISA kit from R&D Systems (Minneapolis, MN, USA) as a comparator. In both cases, the ELISAs consist of sandwich format 96-well plate assays using a monoclonal–polyclonal (directly conjugated) format in the case of the R&D assay compared with a monoclonal-biotinylated monoclonal format with additional streptavidin-horseradish peroxidase conjugate in the case of the Siemens assay. Samples were assayed in duplicate according to the manufacturers' protocols. The sensitivities (Limit of Detection) of the Siemens and R&D assays as quoted by the manufacturers are 2.5 and 2.28 pg/mL with a standard curve range up to 750 (all standards in the Siemens assay have been prediluted 2-fold similar to the recommended sample dilution and allowing direct readout of sample results without correction for dilution – for example, the top standard in the Siemens assay is actually labelled as 1500 pg/mL) and 1000 pg/mL respectively. Assay-specific quality control (QC) samples were run on each plate.

### Assay validation

#### Comparison of EDTA plasma versus serum

The original intention was to validate the Siemens assay for use with EDTA plasma as our sample of choice but we first investigated if similar results were obtained with both serum and plasma. Matched pair EDTA plasma and serum samples (*n* = 15; 8 RCCs, 7 control) were analysed using the Siemens assay and subsequently the R&D Systems assay.

#### Comparison of assay standardization

Standards provided with the Siemens kit were assayed on the R&D ELISA using the appropriate diluents and the concentrations determined. The converse analysis was also undertaken. The range of the standards used in each case was different and determined by the differing ranges of the two ELISAs. In addition, purified recombinant CA IX (R&D Systems) was also measured using both assays.

#### Intra- and inter-assay precision

For each assay, intra-assay precision was determined by assessing three QC samples covering the high, mid and low range of the standard curves six times across a plate. Inter-assay precision was determined by assessing each QC sample across three different plates with duplicate wells for each determination.

#### Parallelism assessment

Two serum samples and three plasma samples (R&D assay) and two plasma and one serum sample (Siemens assay) were serially doubly diluted a further three times over and above the initial normal dilution for analysis using the appropriate dilution buffer for each assay and assessed for parallelism against the standard curves. For the Siemens assay, a further three EDTA plasma samples were also later tested in the presence of excess EDTA (+10 mg/mL final additional concentration) following spiking of the samples with stock EDTA.

#### Recovery assessment

Stock solutions of recombinant CA IX (rCA IX) in 0.1% w/v HSA (Sigma-Aldrich, Poole, UK) were prepared using the appropriate assay sample diluents to achieve concentrations 10-fold higher than the required final spike concentrations (see below). rCA IX was spiked into samples at 10% of sample volume and the results were compared with samples spiked with diluent alone. Five matched pairs of serum and plasma samples were examined in the R&D assay. For the Siemens assay three EDTA plasma samples only were used, as at this time the issues with using serum in this assay had been identified. Additionally, recovery was examined in the presence of excess EDTA (11.8 mg/mL) to maximize removal of calcium. Spike concentrations were determined taking into account the different standardization of each assay. Low spikes were aimed at providing an increase in CA IX concentration of 100 pg/mL in the Siemens assay and 200 or 250 pg/mL in the R&D assay. High spikes were aimed at providing an increase in CA IX concentration of 400 pg/mL in the Siemens assay and 600 or 750 pg/mL in the R&D assay. Absolute concentrations of the spikes used for the recovery calculations were also checked by spiking into diluent buffer and assaying in parallel. Percentage recovery was calculated as (final concentration − initial concentration)/added concentration) × 100.

#### Specificity

The specificity in terms of cross-reactivity with other members of the CA family was assessed by analysing recombinant CA II and CA XII at 1500 pg/mL in both assays.

### Investigation of the effect of metal ions on CA IX measurement

To investigate the apparent differences in results obtained between EDTA plasma and serum in the Siemens assay, stock CaCl_2_ and MgCl_2_ solutions (400 mmol/L) and dipotassium EDTA (36, 50 and 100 mg/mL) were prepared using Milli-Q water. CaCl_2_ (and subsequently MgCl_2_ in some cases) solutions were spiked (1 in 20) to a final concentration of +20 mmol/L into an RCC serum sample and three RCC EDTA plasma samples (to generate ‘serum’ *in vitro* from plasma – plasma treated in this way clotted and was centrifuged at 1000***g*** for 10 min to separate the resultant ‘serum’ from the clot). Conversely, EDTA (36 mg/mL) was spiked (1 in 20) to a final concentration of 1.8 mg/mL into a serum sample and analysed. The degree of saturation of the effect of EDTA contained within the EDTA blood tubes was also investigated by adding further EDTA (+10 mg/mL) to EDTA plasma samples (4 normal and 6 RCCs). The reversibility of the effects of EDTA/metal ions was also checked by spiking EDTA plasma as above with CaCl_2_ to promote clotting followed by addition of EDTA. All samples were compared with samples spiked with Milli-Q water. These experiments were subsequently repeated using recombinant CA IX where stock solutions of 250–500 pg/mL rCA IX were spiked with EDTA (final concentration +1.8 mg/mL) or CaCl_2_ as above.

### Determination of which antibody–antigen interaction is affected by metal ions

In view of the results showing a metal ion-dependence of CA IX measurement in the Siemens assay, a crossover experiment was undertaken to determine which antibody was responsible for this effect. Assays were run in parallel for both R&D and Siemens assays using six columns of the 12-column format kits in each case. The first four columns in each case contained the appropriate standard curves, QC samples and four matched serum/EDTA plasma samples, all examined in duplicate. For the next two columns of the plate, the capture antibody wells were exchanged between the assays and the same four matched pairs of serum/EDTA plasma samples analysed. Preliminary analyses had been undertaken to show that the capture antibodies could be exchanged between kits in this way with minimal effect on the assay background and compatibility in terms of the new antibody pairs still allowing detection of CA IX and producing absorbance signals within the working range of the assay.

### Data analysis

Analysis of replicates and inter-/intra-assay precision was performed by calculating the coefficient of variation (%CV). The significance of the differences between paired plasma and serum samples was performed using paired Student's *t*-test. Parallelism was determined by back calculating the concentration of the individual dilutions and assessing the %CV between the four back-calculated concentrations (pass criteria <15%).^28^ Statistical analyses were conducted using SPSS (v16.0) and GraphPad Prism (v5) software.

## Results

### Assay validation

**Figure 1:**
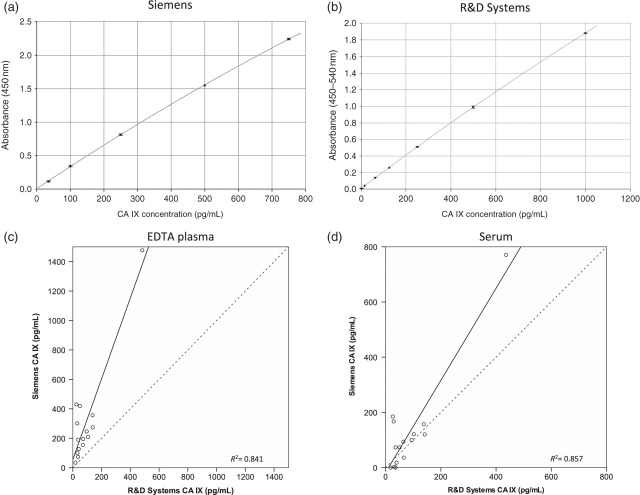
Representative examples of standard curves for the (a) Siemens (corrected for pre-dilution) and (b) R&D assays. (c) and (d) Relationship between concentrations of CA IX in 15 matched pairs of EDTA plasma or serum, respectively, using these two assays. The dotted line in each case is the line of equivalence. CA, carbonic anhydrase

Typical standard curves are shown (Figures 1a and b). Analysis of rCA IX on both assays showed a ratio of Siemens:R&D values obtained of 2.8. In support of this indicating differences in standardization, analysis of five Siemens standards (35–750 pg/mL) on the R&D assay showed concentrations ranging from 12.0 to 236.1 pg/mL, respectively, with an average ratio of Siemens:R&D values of 3.13 (SD 0.17). Conversely, a reciprocal analysis analysing three R&D standards (62.5–250 pg/mL assigned concentrations) on the Siemens assay showed concentrations ranging from 154.7 to 626.5 pg/mL, respectively, with an average ratio of Siemens:R&D values of 2.51 (SD 0.03). These support the difference seen with the purified recombinant protein with slight differences presumably due to differing backgrounds with the different standards across the two assays. However, subsequent comparison of 15 plasma samples across both assays showed no such simple relationship between assay results with Siemens:R&D ratios varying from 1.95 to 17.3. Similarly, comparison of the 15 matched serum samples across both assays showed ratios varying from 0 to 7.1 (Figures 1c and d). These results support a difference in standardization between assays but also indicate that with clinical samples there are other potential factors affecting the results.

**Figure 2:**
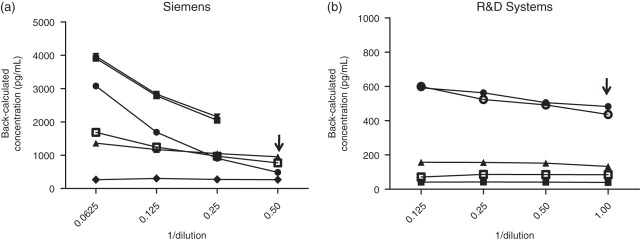
Assessment of parallelism in the (a) Siemens and (b) R&D ELISAs for EDTA plasma and serum samples (open symbols = serum; closed symbols = plasma) by comparing the back-calculated concentrations against serial doubling dilutions of the samples. The arrow in each case indicates the normal working dilution used in the assay. ELISA, enzyme-linked immunosorbent assay

Intra-and inter-assay precision was acceptable for both assays at <10% with the exception of the lowest concentration controls on the Siemens assay, which exhibited CVs of 14.5% and 18.6%, respectively (Table 1). Generally for both assays the SD of the duplicates for any sample was <5% of the mean result. The criteria for assessing parallelism are described above in the Data analysis section. Using these criteria, the three samples initially tested on the Siemens assay failed to dilute out in parallel (Figure 2a). When one of these plus a further two plasma samples were tested in the presence of excess EDTA, this was still the case with one of the three passing. For the R&D assay, one sample had extremely low concentrations of CA IX and was unsuitable but the remaining two serum and three EDTA plasma samples all passed on parallelism, three of which were among the samples analysed on the Siemens assay (Figure 2b).

**Table 1:** Intra- and inter-assay precision for the Siemens and R&D Systems ELISAs for carbonic anhydrase IX

	Intra-assay precision				Inter-assay precision			
Assay	Mean conc. (pg/mL)	*n*	SD	CV (%)	Mean conc. (pg/mL)	*n*	conc. SD	CV (%)
Siemens	136.0	6	19.7	14.5	145.9	3	27.1	18.6
	284.8	6	20.4	7.2	289.7	3	25.7	8.9
	728.0	6	29.1	4.0	742.8	3	36.7	4.9
R&D	150.5	6	5.8	3.8	140.1	3	11.7	8.4
	288.8	6	21.6	7.5	271.4	3	22.4	8.3
	548.4	6	28.5	5.2	523.5	3	42.8	8.2

ELISA, enzyme-linked immunosorbent assay; CV, coefficient of variation

The intra-assay results were derived from analysis of six replicates of each QC sample with the inter-assay results being based on three separate measurements each in duplicate

Recovery was acceptable (within the range 80–120%) for all EDTA plasma samples tested on the Siemens assay and on the R&D assay (Table 2). However, one of five serum samples failed in recovery (<80%) of both high and low spikes on the R&D assay. Cross-reactivity with CA II and CA XII was minimal/non-existent when tested using stock solutions of 1500 pg/mL, producing results of 3.6 and 10.5 pg/mL, respectively, on the Siemens assay and undetectable and 3.4 pg/mL on the R&D assay.

**Table 2:** Recovery of recombinant carbonic anhydrase (CA) IX spiked into EDTA plasma and serum samples and measured using either the Siemens or R&D assays

		EDTA plasma			Serum		
Assay	Sample	Initial CA IX (pg/mL)	% recovery (low spike)	% recovery (high spike)	Initial CA IX (pg/mL)	% recovery (low spike)	% recovery (high spike)
Siemens	1	516.5	102.2	88.4			
	2	830.9	93.3	81.0			
	3	485.1	94.1	88.2			
R&D	4	483.3	81.7	80.2	502.4	59.4	66.7
	5	92.4	82.3	86.3	92.7	86.7	84.9
	6	26.9	81.7	83.0	27.4	81.5	91.2
	7	47.6	82.0	91.5	53.2	89.9	93.5
	8	84.0	97.8	90.7	88.0	94.0	86.6

### Measurement of CA IX

**Figure 3:**
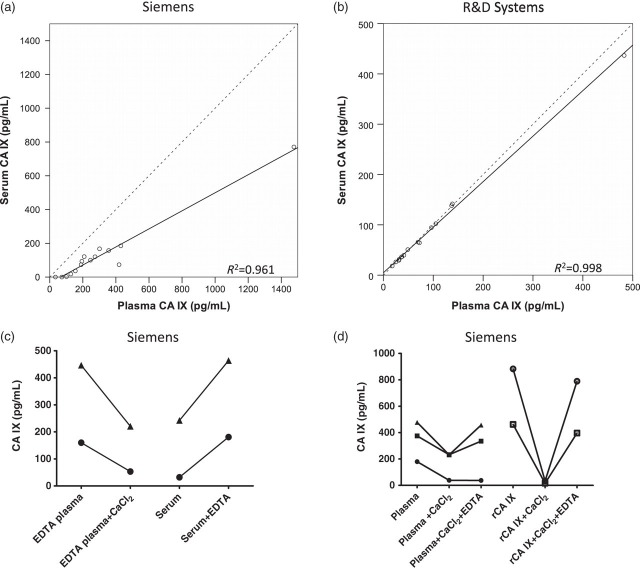
Graphs showing the relationship between concentrations of CA IX in EDTA plasma or serum (*n* = 15 pairs) assessed using either the (a) Siemens or (b) R&D assays. The dotted line shows the line of equivalence with a slope of 1. (c) Effects of adding 20 mmol/L CaCl_2_ to EDTA plasma or of adding 1.8 mg/mL EDTA to serum on the CA IX concentrations as measured using the Siemens assay. (d) The reversibility of the effect is shown by sequential addition of CaCl_2_ and EDTA to EDTA plasma or recombinant CA IX. CA, carbonic anhydrase; rCA, recombinant CA

Comparison of EDTA plasma and serum concentrations of CA IX in 15 matched pair samples showed significantly higher concentrations in EDTA plasma samples compared with serum (*P* < 0.001) when measured using the Siemens assay and although a significant correlation was seen (*P* < 0.001; *r^2^* = 0.961), the slope of the line was only 0.538 with several samples lying distantly (Figure 3a). Using the Siemens assay, concentrations in EDTA plasma ranged from 34.5 to 1476.4 pg/mL (mean 305.8 pg/mL), while concentrations in serum were <2.5 to 770.6 pg/mL (mean 127.9 pg/mL). In contrast, no significant difference between the two sample types was found using the R&D assay and a significant correlation (*P* < 0.001; *r^2^* = 0.998) and a slope of 0.905 were seen (Figure 3b). Using the R&D assay, concentrations in EDTA plasma were 17.7 to 482.9 pg/mL (mean 91.3 pg/mL) and concentrations in serum were 18.2 to 436.6 pg/mL (mean 87.4 pg/mL).

### Effect of metal ions

As initially we were only using the Siemens assay, first investigations focused on clotting events causing the apparent reduction in measurable CA IX in serum rather than this being assay-specific. Spiking of EDTA plasma with excess calcium, which resulted in clot formation and therefore formation of serum *in vitro*, did result in a reduction in CA IX concentrations detected almost to those found in matched serum samples (*n* = 3; mean 172.0, SD 75.6 versus mean 83.7, SD 55.8 pg/mL; *P* = 0.036) supporting this possibility (Figure 3c). However, addition of EDTA to serum produced a marked elevation of CA IX detected (Figure 3d) and this apparent reversibility of the changes was completely demonstrated in two of the three samples when addition of calcium to plasma was followed by addition of EDTA (Figure 3d). This was also seen with recombinant CA IX. These results clearly show that clotting *per se* did not account for the differences but that metal ions may be the cause. Magnesium produced similar effects to calcium and importantly the concentration of EDTA in plasma from the collection tube was found not to be present in sufficient excess, as further addition of EDTA (+10 mg/mL) resulted in further increases of CA IX detected (*P* < 0.038; *n* = 7), although little difference was seen between +5 and +10 mg/mL. As expected, no effects of calcium or EDTA addition were seen in the R&D assay where differences in plasma and serum were not observed, which also supports this explanation.

**Figure 4:**
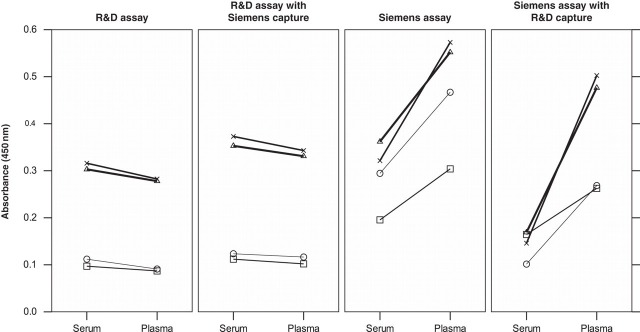
Effect of crossing over antibodies between assays to determine which antibody–antigen interaction accounts for the metal-ion-dependent effects seen in the measurement of CA IX. Results are shown for four matched pairs of EDTA plasma and serum samples assayed using either the R&D assay, the Siemens assay or each of these but with the capture antibodies swapped between the assays. Absorbances were measured at 450 nm in both cases but background subtraction at 540 nm carried out in the case of the R&D systems assay. CA, carbonic anhydrase

When capture antibodies were exchanged between assays, it was very apparent that introduction of the Siemens capture antibody into the R&D assay maintained the absence of difference in absorbance values in plasma versus serum. However, pairing the R&D capture antibody with the Siemens detection antibody resulted in marked differences in absorbance values for the two fluid types as seen in the Siemens assay (Figure 4). This clearly indicates that the Siemens detection antibody i.e. the M75 clone^29^ is responsible for the differences seen in CA IX in plasma and serum and together with the previous results is consistent with the recognition by this antibody of an epitope which is metal-ion dependent.

## Discussion

There is a substantial need for new biomarkers in RCCs and CA IX is of potential interest. Both plasma and serum concentrations of soluble CA IX are reportedly elevated in RCCs, although reports vary with both a positive correlation with tumour size and no correlation being reported and a decrease postsurgery in some patients.^20,21,23^ A study of 91 patients with conventional RCCs found that serum CA IX was significantly associated with recurrence-free survival.^19^ However, a recent study of serum samples from 287 RCC patients failed to find a significant prognostic association with soluble CA IX, although it was positively correlated with stage and higher concentrations were also found in clear cell compared with papillary subtypes.^26^ Analysis of samples from a subset of 66 patients with advanced RCCs randomized to either sorafenib treatment or placebo (TARGET) reported that plasma CA IX was prognostic for overall survival although not of independent significance, and was unchanged and not associated with benefit from treatment.^27^

No studies have described any validation of the assays used to measure CA IX and clearly we show that there are marked differences in the performances of two commercially available ELISAs, with one in particular (Siemens) generating different results depending on whether EDTA plasma or serum was used. Results in the manufacturer's kit insert showed a possible trend, although non-significant and not as marked as reported here but only 10 samples were examined and all from healthy volunteers. Our results clearly show that the R&D Systems ELISA is suitable for use with EDTA plasma and serum and performs acceptably, although further recovery studies, confirmation of the sensitivity and examination of other relevant preanalytical factors are needed. In contrast, although the Siemens ELISA passed several elements of the validation assessment, there were issues with lack of parallelism which to some extent may be due to our finding of the importance of metal ions on the concentration of CA IX measured. This latter effect meant that by adding calcium or removing metal ions through the use of chelating agents such as EDTA, the concentration detected could be varied reversibly. Although the calcium concentration used here was excess rather than physiological to promote clotting, it is likely that variations in the physiological range could influence the measurement still as further addition of calcium to serum resulted in further reductions. Similarly, the amount of EDTA present in blood collection tubes as an anticoagulant did not appear to have saturated the chelating effect, implying that even in EDTA plasma accurate determination was not guaranteed. Clearly, variability in filling of the blood tube at venepuncture with consequent effects on final EDTA concentration would also contribute to further variation.

At least two forms of soluble CA IX have been described (50 and 54 kDa) formed by cleavage of the extracellular region of CA IX.^20^ This region contains a negatively charged N-terminal proteoglycan-like (PG) domain and the CA catalytic domain, which has three metal binding regions to which zinc is known to bind and which is essential for the catalytic activity.^30^ The M75 detection antibody in the Siemens assay recognizes a linear epitope (containing the repeat motif of GEEDLP) on the PG domain^31^ whereas the V10 capture antibody targets a conformational epitope on the CA domain.^6,32^ Recent evidence also supports the binding of multiple divalent cations to the highly negatively charged PG domain with an effect on the catalytic activity of CA IX.^5^ Our study provides further indirect evidence for this as the M75 antibody, which binds to this domain and which appears to be responsible for the effects seen in our study with plasma and serum, appears to bind to an epitope which is metal ion-dependent. These effects may be mediated by direct inhibition of M75 competitively or through induction of a conformational change.

Several of the previously discussed studies have used the R&D Systems assay and under conditions that are likely to have produced robust results. However, several studies in renal cancer but also in ovarian, lung and bladder cancers have either used the Siemens assay or assays involving the M75 antibody^20,22–25,27^ and the results may therefore be affected as described here. Oncogene Science/Siemens Healthcare Diagnostics Inc. was provided with a summary of the results reported in this paper. The M75 antibody has been used to investigate tissue concentrations of CA IX immunohistochemically and there is the very real possibility that buffers used in such studies may influence the results seen, which warrants further investigation.

Although differences in concentrations of analytes between plasma and serum have been shown previously due to effects of clotting, for example higher serum concentrations of vascular endothelial growth factor due to release by platelets^33^ or lower serum concentrations of osteopontin due to cleavage during clotting,^34^ the phenomenon described here where differences in measured levels are due to metal ion-dependence of the specific epitope recognized by an antibody in an assay is much less well described. Examples include the calcium dependence of calretinin measurements possibly analogous to this study,^35^ elimination of false-positive results in the RSR GADAb ELISA by adding calcium to EDTA plasma possibly by effects on proteases^36^ and the lower concentrations of S100A12 in EDTA plasma possibly due to loss of oligomerization and consequent antibody binding in the absence of calcium.^37^ Such studies illustrate the need for careful independent validation of commercially available immunoassays prior to use in research studies to avoid erroneous conclusions regarding potential clinical utility and wasted resources.

## DECLARATIONS

**Competing interests**: None of the authors have any competing interests.

**Funding**: This work was funded by a National Institute of Health Research Programme (RP-PG-0707-10101) and Cancer Research UK (C8175/A12312).

**Ethical approval**: Ethical approval was obtained from Leeds (East) Research Ethics Committee (98/045).

**Guarantor**: REB.

**Contributorship**: All authors contributed to the design of the study. REB and DT provided direct supervision with TCW and MPM carrying out the lab work and REB, DT, TCW and MPM all contributed to data analysis. Initial drafts of the paper were done by TCW and MPM with REB, DT and PJS revising, editing and approving the final manuscript. TCW and MPM are joint first authors.

**Acknowledgements**: We are grateful to the patients for donating their samples to our research studies, staff within the research and clinical teams at St James's University Hospital for help in obtaining and processing samples and Cancer Research UK and National Institute of Health Research for funding this research. We are also grateful to Siemens Healthcare Diagnostics for their contributions towards the ELISA kits used in this study.
